# Identification of an Interstitial 18p11.32-p11.31 Duplication Including the *EMILIN2* Gene in a Family with Porokeratosis of Mibelli

**DOI:** 10.1371/journal.pone.0061311

**Published:** 2013-04-10

**Authors:** Corrado Occella, Dario Bleidl, Paolo Nozza, Samantha Mascelli, Alessandro Raso, Giorgio Gimelli, Stefania Gimelli, Elisa Tassano

**Affiliations:** 1 Unità Operativa di Dermatologia, Istituto Giannina Gaslini, Genova, Italy; 2 Unità Operativa di Anatomia Patologica, Istituto Giannina Gaslini, Genova, Italy; 3 Unità Operativa di Neurochirurgia, Istituto Giannina Gaslini, Genova, Italy; 4 Unità Operativa Semplice, Laboratorio di Citogenetica, Istituto Giannina Gaslini, Genova, Italy; 5 Service of Genetic Medicine, University Hospitals of Geneva, Geneva, Switzerland; University of Tennessee, United States of America

## Abstract

Porokeratosis is a rare disease of epidermal keratinization characterized by the histopathological feature of the cornoid lamella, a column of tightly fitted parakeratocytic cells, whose etiology is still unclear. Porokeratosis of Mibelli is a subtype of porokeratosis presenting a single plaque or a small number of plaques of variable size located unilaterally on limbs. It frequently appears in childhood and occurs with a higher incidence in males. Cytogenetic analyses were performed in all members of the family on lesioned and uninvolved skin. An array-CGH analysis was also performed utilizing the Human Genome CGH Microarray Kit G3 400 with 5.3 KB overall median probe spacing. Gene expression was performed on skin fibroblasts. In this study, we describe a Caucasian healthy 4-year-old child and his father showing features of porokeratosis of Mibelli. Array-CGH analysis revealed an interstitial 429.5 Kb duplication of chromosome 18p11.32-p11.3 containing four genes, namely: *SMCHD1, EMILIN2, LPIN2*, and *MYOM1* both in patient and his father. *EMILIN2* resulted overexpressed on skin fibroblasts. Also other members of this family, without evident signs of porokeratosis, carried the same duplication. Among these genes, we focused our attention on elastin microfibril interfacer 2 (*EMILIN2*) gene. Apoptosis plays a fundamental role in maintaining epidermal homeostasis, balancing keratinocytes proliferation, and forming the stratum corneum. *EMILIN2* is known to trigger the apoptosis of different cell lines negatively affecting cell survival. It is expressed in the skin. We could speculate that the duplication and overexpression of *EMILIN2* cause an abnormal apoptosis of epidermal keratinocytes and alter the process of keratinization, even if other epigenetic and genetic factors could also be involved. Our results could contribute to a better understanding of the pathogenesis of porokeratosis of Mibelli.

## Introduction

Porokeratosis (PK) is a heterogeneous group of disorders of epidermal keratinization characterized by atrophic patches surrounded by a stack of tightly fitted parakeratotic cells called the *cornoid lamella*, which is the histopathological feature for this group of disorders. Different clinical variants of porokeratosis have been recognized, each with its own specific properties in terms of morphology, distribution, and clinical course, namely: porokeratosis of Mibelli (PM), disseminated superficial porokeratosis (DSP), disseminated superficial actinic porokeratosis (DSAP), porokeratosis palmaris et plantaris disseminated (PPPD) and linear porokeratosis (LP)[1].

PM consists of a single plaque or a small number of plaques of variable size, usually located unilaterally on limbs. It frequently appears in childhood but may appear at any age, especially in nonhereditary cases, with a higher incidence in males. DSP is a variant of PM characterized by small erythematous or pigmented keratotic papules with central atrophy, located on the trunk, genitals, palms, and soles [2]. The aetiology of porokeratosis is still unclear. An autosomal dominant inheritance has been established for PM, DSP, DSAP, and PPPD [3]–[5]. A locus for DSP has been described, by linkage analysis, to map to chromosome 18p11.3 with a peak locus to a 2.7 Mb region [6]–[7]. Mutation analysis of twelve candidate genes mapped in this region has brought to negative results. Interestingly, this region overlaps a novel minor psoriasis susceptibility locus mapped on 18p11.23 in Finnish families, and two independent studies of gene expression profiling of porokeratosis showed a striking similarity between the gene expression profiles of porokeratosis and psoriasis [8]–[10]. In addition, both studies showed the implication of a number of upregulated genes in porokeratosis. These genes are involved in epidermal differentiation, intercellular communication, and immune response. Recently, a critical region of 38 Mb on chromosome 12q21.2–24.21 has been identified as a probable second locus for DSP [11].

Here we describe a young male and his father showing features of porokeratosis of Mibelli and a 429 Kb interstitial duplication of chromosome 18p11.32-p11.31.

## Results

### Clinical report

**Figure 1:**
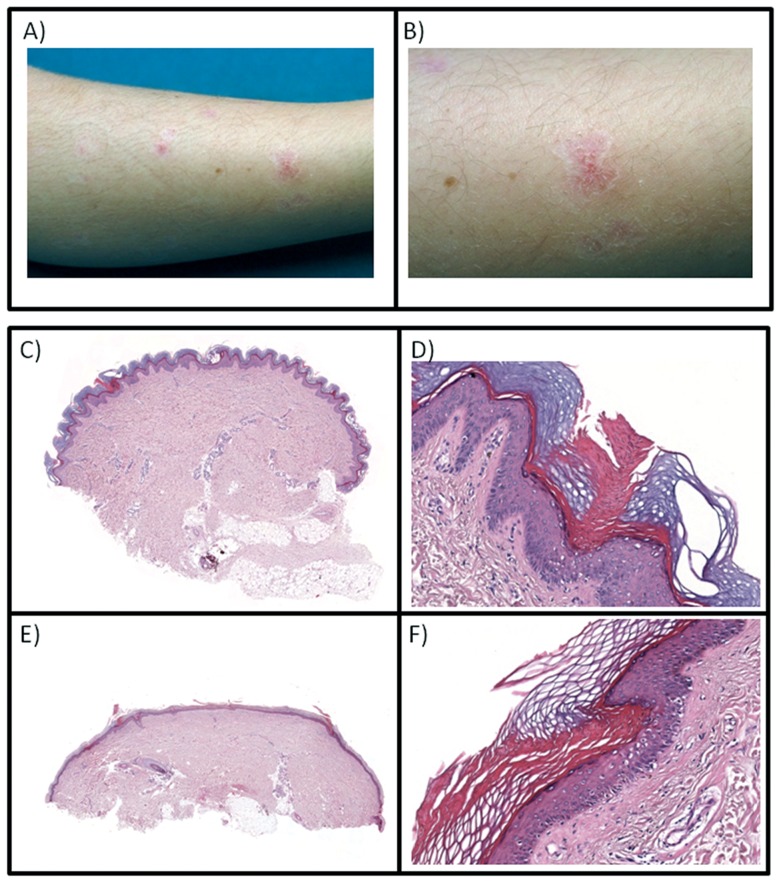
Clinical images. A) Clinical image revealing a whitish-red plaque on the lower right leg of the 4-year-old proband. The affected area is made up of numerous whitish-red round papules that coalesce into irregular plaque and single papules, the area perimeter defined by a whitish border and cleaved by a central furrow. Slightly raised whitish-red portions can also be observed. B) Enlarged detail of the lesion. C) The patient's skin biopsy shows slight papillomatosis and ortokeratosis of epidermis and a cornoid lamella; the derma appears normal (H&E, 10×). D) A column of parakeratotic cells makes up the cornoid lamella (H&E, 200×). E) The examination of the skin biopsy of the father shows atrophic epidermis with two cornoid lamellae; solar elastosis and sparse perivascular lymphocytic infiltrate can be recognized (H&E, 10×). F) The cornoid lamella is very thin (H&E, 200×).

A Caucasian healthy 4-year-old child presented annular plaques with central atrophy on his right lower leg that first appeared two years earlier. The patient's clinical history and physical exam suggested porokeratosis of Mibelli. Clinical examination revealed whitish-red round papules of 1–3 mm in diameter that coalesced into an irregular plaque and single papules, the overall patch extending 3.5 cm in length. The area of plaque and papules had an annular appearance with whitish borders. Annular plaques presented with central atrophy and elevated keratotic borders that had a longitudinal furrow, with slightly raised whitish-red portions on either side of the furrow (Fig. 1, A, B). Also the patient's 36 years-old father showed very similar lesions at the inferior extremities present for several years, never investigated.

### Histology

Histological examination of skin biopsy of the propositus showed slight papillomatosis and ortokeratosis of epidermis and a cornoid lamella (Fig. 1, C, D), while his father showed atrophic epidermis with two cornoid lamellae, solar elastosis, and sparse perivascular lymphocytic infiltrate (Fig. 1, E, F).

### Cytogenetics and array CGH analysis

**Figure 2:**
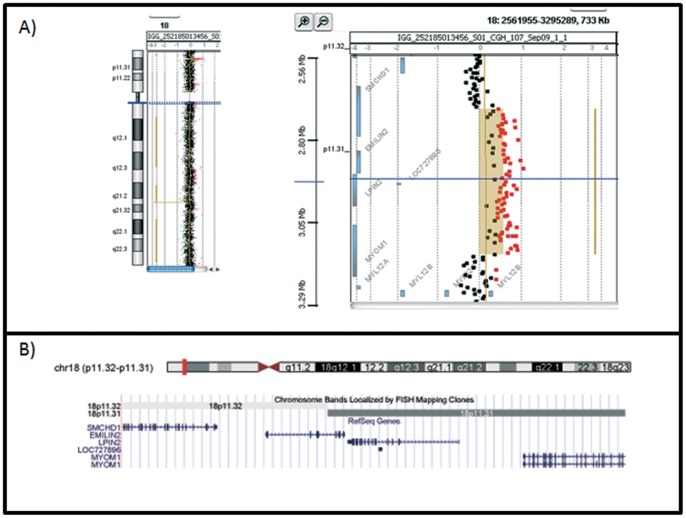
Array-CGH results. A) Result of array-CGH analysis of chromosome 18 with Agilent Human Genome CGH microarray Kit G3 400K. The 18p11.32p11.31 duplicated region extends between oligomers A_16_P20755613 (2,724,439 bp) and A_16_P40933037 (3,153,981 bp) B) Gene contents of the duplicated region.

**Figure 3:**
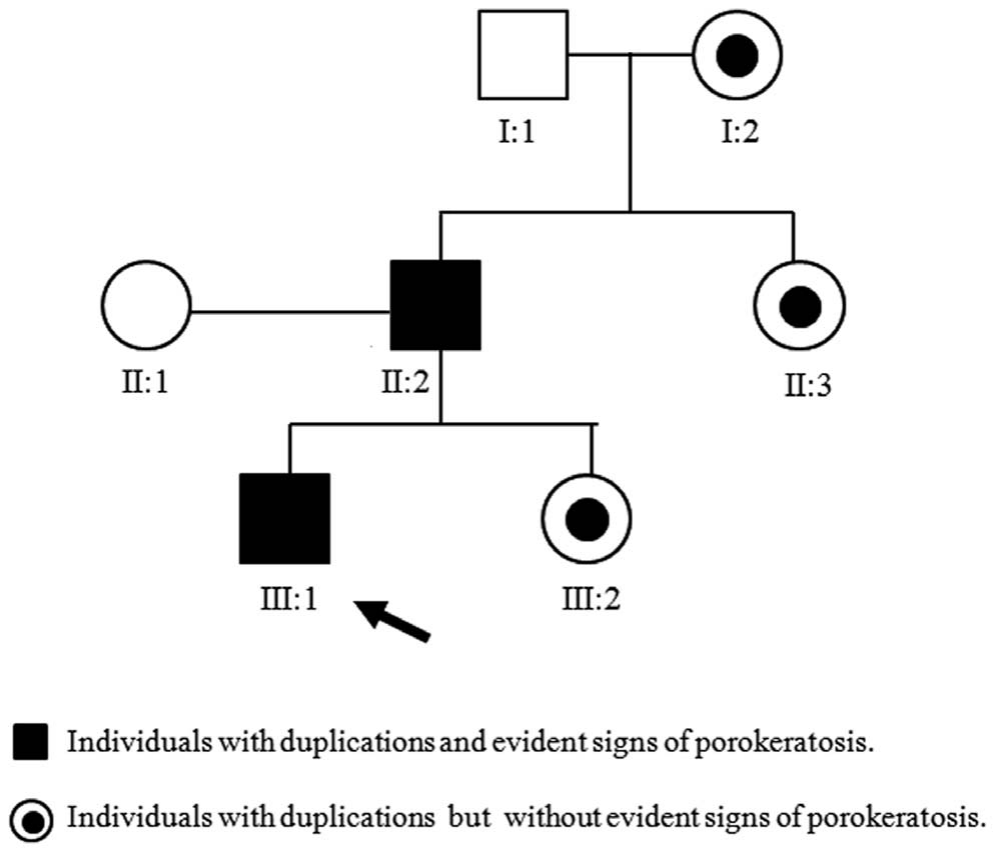
Pedigree of the family. Full symbols (black) indicate the individuals with duplication and clear signs of porokeratosis. Symbols with black dots indicate individuals with duplication but without evident signs of porokeratosis. White symbols indicate normal individuals.

Cytogenetic investigations performed on peripheral blood lymphocytes in all members of the family showed a normal karyotype. The karyotype of fibroblasts from affected and uninvolved skin of the father was normal and no chromosomal breakages were found. To investigate the genomic DNA of our patient and his father for submicroscopic aberrations, we performed array-CGH analyses, using the Agilent G3 400 Kit. An interstitial 429.5 Kb duplication of chromosome 18p11.32-p11.3 from A_16_P20755613 (2,724,439 bp) to A_16_P40933037 (3,153,981 bp) oligomers was showed (Fig. 2). Also other members of this family carried the same duplication but without evident signs of porokeratosis (Fig. 3). In particular, the little sister of our proband had only six months of age and she still showed no signs because porokeratosis of Mibelli occurs during childhood [12]. The paternal grandmother, and the paternal aunt reported that they did not have signs of porokeratosis but were not subjected to thorough examination by an expert dermatologist. Furthermore, we need to bear in mind that the disease is more frequent in males. The high diversity of clinical presentation could be also caused by the influence of interactions between genetic and environmental factors on clinical manifestation or differential environmental exposures experienced by different individuals.

The duplicated region contains four genes: *SMCHD1* (NM_015295.2), *EMILIN2* (elastin microfibril interfacer 2; MIM 608928), *LPIN2* (lipin 2; MIM 605519), *MYOM1*(myomesin 1; MIM 603508) according to UCSC GRCh 37/hg19 assembly (http://genome.cse.ucsc.edu/) (Fig. 2, B).

None 18p11.32p11.31 duplication was detected in two control groups consisting of 3645 individuals and in the Database of Genomic Variants (http://projects.tcag.ca/) [13]–[14].

### Expression analyses

**Figure 4:**
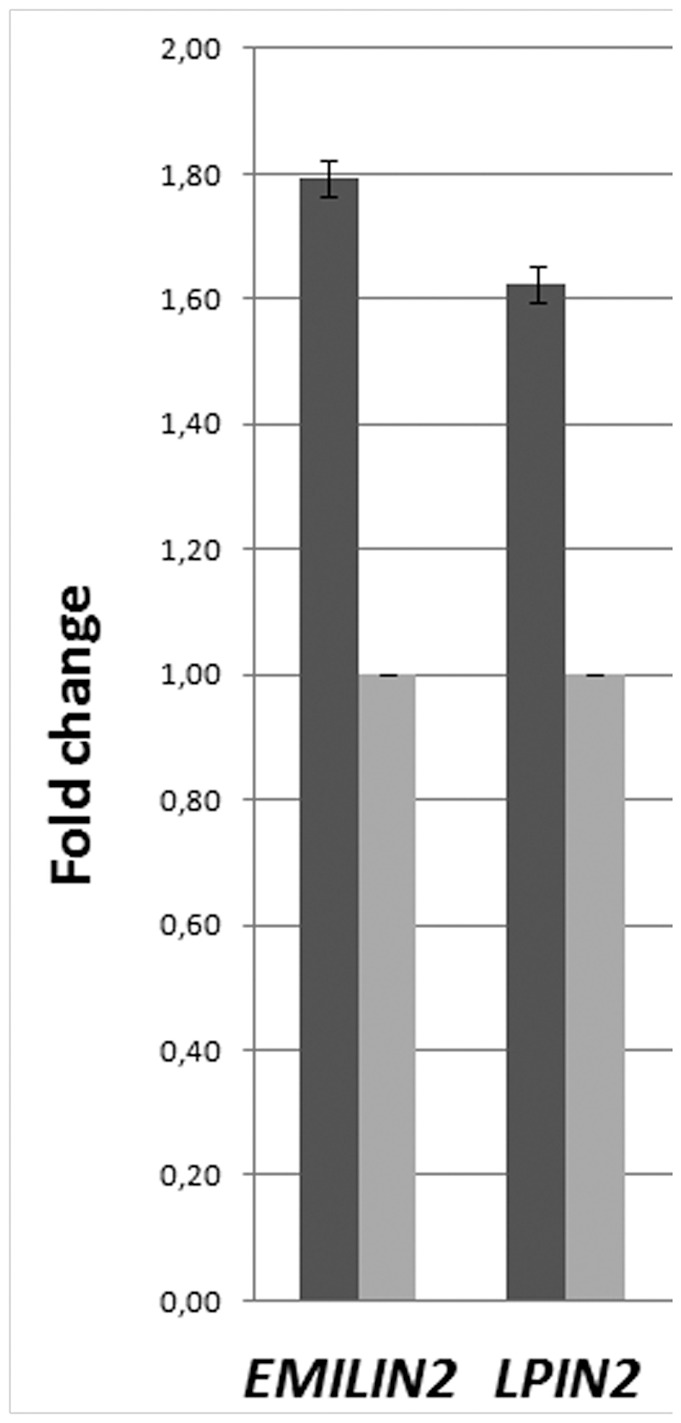
Expression studies. Comparison *of EMILIN2, LPIN2*, and *MAD1L1* expression in the primary skin fibroblast cultures of proband's father compared with controls (Ct). The histogram shows the median of values: dark grey corresponds to patient's fibroblasts (#102) normalized to the median of values of six normal control's fibroblasts (light grey).

The expression of the candidate genes involved in the duplication was determined by qRT-PCR (Fig. 4). A significant fold change of expression of the genes on 18p11.3 was observed in the primary fibroblast cultures of the patient's skin as compared to controls. The median gene expression of *EMILIN2* and *LPIN2* in patient's fibroblast was 1.79 and 1.62-fold higher than in normal fibroblasts, respectively.

## Discussion

Porokeratosis is a rare and heterogeneous disorder of epidermal keratinization showing a clinical variety. Different variants of porokeratosis (PK) have been subsequently recognized, each with its own specific properties in terms of morphology, distribution, and clinical course. Porokeratosis of Mibelli (PM) and disseminated superficial porokeratosis (DSP) are clinical variants that appear in childhood. Our patients were diagnosed with porokeratosis of Mibelli on the basis of the number, size and distribution of plaques, as well as age of onset. In fact, hereditary PM appears in childhood and occurs with higher incidence in males [12]. We found very diverse clinical manifestations of the disease among our patients carrying the same duplication. The affected family members (II, 2; father) and (III, 1; proband) showed typical clinical features. The sister of our propositus (III, 2) did not show the typical lesions probably because she was only six months of age. As referred, the individuals (I, 2) grandmother and (II, 3) paternal aunt of the proband probably showed an extremely mild phenotype but they have never been visited by an expert dermatologist. The diversity in clinical presentation among these cases, carrying the same duplication, could be caused by the influence of interactions between genetic factors on clinical manifestation or by differential enviromental exposures. Zhang et al. [15] performed an exome sequencing study in individuals affected by DSAP and he identified mutations in *MVK* gene. Similarly, they found very diverse clinical manifestations of disease phenotype among those individuals carrying the same pathogenic mutations, ranging from an extremely mild phenotype to typical clinical features. Our patient and the other family members showed a 429.5 Kb duplication of a segment overlapping 18p11.32 and 18p11.31 bands, containing four genes: *SMCHD1, EMILIN2, LPIN2*, and, *MYOM1*. Among these genes, we focused our attention on *EMILIN2* gene which seemed to be particularly interesting. *EMILIN2* gene encodes a glycoprotein of the extracellular matrix, whose expression has been found in a variety of tissues during mouse development, suggesting its important role in organogenesis [16]. In Zebrafish embryo it was expressed in the dermis of trunk and tail [17]. Recently, it has been demonstrated that *EMILIN2* promotes apoptosis in different cell lines binding directly to death receptors DR4 and DR5 (the receptors for TNF-related apoptosis-inducing ligand) subsequently activating them [18]. The direct interaction of an extracellular matrix (ECM) protein with death receptors represents a new mechanism where ECM cues can negatively affect cell survival by activating an extrinsic apoptotic pathway. Apoptosis plays a fundamental role in maintaining epidermal homeostasis, balancing the keratinocyte proliferation, and in forming the stratum corneum; it is also be able to eliminate pre-malignant cells. It is known that DR4 and DR5 are expressed by normal keratinocytes and *EMILIN 2* is expressed in the skin [18]–[20]. Studies on apoptosis in seven patients with porokeratosis concluded that an abnormal early apoptosis of keratinocytes accompanied by dysregulation of terminal differentiation may be involved in the pathogenesis of porokeratosis [20]. Mongiat et al. [18] demonstrated that knockdown of *EMILIN2* increased tumor cell survival, while overexpression impaired tumor cell growth in vitro. Moreover, an increased expression of the p53 tumour suppressor gene product has been found in keratinocytes under or adjacent to the cornoid lamella in all subtypes of porokeratosis [21].

Surely, a dysregulated cutaneous immune response plays an important pathogenetic role in the porokeratosis. In fact, following the detection of helper T cells and Langerhans cells in PM, an involvement of immunological mechanisms has been suggested [22]. Local or systemic changes in immune function could induce decreased immune surveillance, which in turn would prevent pathologic keratinocyte clones from being recognized and immunologically rejected [23].However, it could directly trigger the development and proliferation of a mutant clone of keratinocytes. At the same time, a local immunosuppression could explain the promoting effect of UV rays.

Our study is the first to associate a genetic anomaly and a possible candidate gene for porokeratosis of Mibelli. We could speculate that, at least in our family, the duplication of *EMILIN2* gene may cause an excessive death receptors activation in the skin and an abnormal apoptosis of epidermal keratinocytes leading to the alteration of the process of keratinization which is at the basis of porokeratosis.

## Materials and Methods

### Ethics Statement

The current study was performed using peripheral blood and skin biopsy of the members of the family treated at the Istituto Giannina Gaslini, Genova, Italy. The parents of the patients gave written informed consent allowing molecular and genetic studies. We didn't request to review our study protocol and approval by Review Board of our institution, because our study request only classical and molecular cytogenetic analyses. For cytogenetics analyses are sufficient only written informed consent of the parents (DM 21 dicembre 2007). The informed consents of the parents were previously approved and authorized by the Review Board of our institution (we don't know the exact date whe the board was consulted about this consent procedure). We didn't conduct research outside our country of residence.

We didn't approach the local authorities before beginning work on this study. The full name of the ethics committee of our institution is Comitato di Etica per la Ricerca Scientifica Biomedica, per la Buona Pratica Clinica e per la Sperimentazione dei Farmaci. I confirm that the only review board that we interacted with regarding this study was the Comitato di Etica per la Ricerca Scientifica Biomedica, per la Buona Pratica Clinica e per la Sperimentazione dei Farmaci. My data are anonymous.

### Cytogenetics and array CGH analyses

Cytogenetic analysis was performed on QFQ-banded metaphases at a resolution of 450–550 bands. Chromosome preparations were made from cultured lymphocytes of the proband and his father. Karyotype analysis was also performed on fibroblasts from a skin biopsy of lesional tissue and uninvolved skin from the proband's father.

Array-CGH was performed on the child (peripheral blood) and his father (peripheral blood, cultured cells from uninvolved skin and lesioned tissue) using Human Genome CGH Microarray Kit G3 400 (Agilent Technologies, Palo Alto, USA) with 5.3 KB overall median probe spacing. CGH analysis was performed in other family members. Labelling and hybridization were performed following the protocols provided by the manufacturers. The array was analyzed with the Agilent scanner and the Feature Extraction software(v8.0). A graphical overview was obtained using the Agilent Genomic Workbench software (Agilent). We have submitted our case to the Decipher database (Patient GGI272544)(https://decipher.sanger.ac.uk/patient/272544).

### Gene expression studies

Total RNA was extracted from 1×10^6^ cells of both primary fibroblast cultures of patient's skin and six fibroblast cultures of healthy donors with the use of Trizol reagent (Invitrogen Life Technologies, Milan, Italy), following the standard procedure. Additionally, RNA underwent silica–cartridge purification using the PureLinkTM system (Invitrogen Life Technologies) and total RNA was treated with (RNAse-free) DNAse. RNA was quantified by Nanodrop (Celbio, Milan, Italy) and its quality and integrity was assessed using Agilent 2100 Bioanalyzer (Agilent, Santa Clara, CA). Double stranded cDNA synthesis was performed using Oligo(dT)_20_ priming by a two-Step cDNA Synthesis kit (Invitrogen Life Technologies).

Expression of the Human genes: *EMILIN2* (NM_032048), *LPIN2* (NM_014646) mapping on chromosomal band 18p11.3 were assessed by quantitative PCR (qPCR) using in-house designed systems following a fine-tuning procedure: *EMILIN2*: forward primer, 5′-GGGCGTTGTCCTCTTTA-3′ and reverse primer, 5′-CGTAGGCGTCTCTCTCG-3′; *LPIN2*: forward primer, 5′- GAGTCCTGAGATCCAAAGAGA-3′ and reverse primer, 5′-CTCCGTTATCACCCAACTTC-3′ [24]. Amplifications were carried out in single plex runs on 25 µL using Express-Sybr GreenER qPCR-SuperMix Universal (Invitrogen, Carlsbad, CA, USA) and gene expression was tested on ABI PRISM 7500 HT Sequence Detection System (Applied Biosystems, Foster City, CA). qPCR efficiencies of each system were calculated using the equation: E = 10−1/slope and data were considered comparable when the difference between the efficiencies was <0.1 [25]. The normalized fluorescent signal was automatically calculated using an algorithm that normalizes the reporter emission signal. Non fluorescent signals were generated by these assays when genomic DNA was used as substrate. The relative quantification of genes transcript was performed according to the comparative method (2^−Δ Δ Ct^, Applied Biosystems User Bulletin no. 2P/N 4303859). Beta actin (ACTB, NM_001101), Pyruvate kinase (PMK2, NM_002654), and Beta-2-microglobulin (B2M, NM_004048) were used as the endogenous control genes for each cell line [24]. The Minimum Information for Publication of Quantitative Real-Time PCR Experiments (MIQE) is provided [26].

## Acknowledgments

We thank the patient's parents for their kind participation and support. We are grateful to the technicians from our laboratories for their skillful help.
